# Modelling Cooperative Tumorigenesis in* Drosophila*

**DOI:** 10.1155/2018/4258387

**Published:** 2018-03-06

**Authors:** Helena E. Richardson, Marta Portela

**Affiliations:** ^1^Department of Biochemistry and Genetics, La Trobe Institute of Molecular Science, La Trobe University, Melbourne, VIC, Australia; ^2^Department of Molecular, Cellular and Developmental Neurobiology, Cajal Institute (CSIC), Avenida Doctor Arce, No. 37, 28002 Madrid, Spain

## Abstract

The development of human metastatic cancer is a multistep process, involving the acquisition of several genetic mutations, tumour heterogeneity, and interactions with the surrounding microenvironment. Due to the complexity of cancer development in mammals, simpler model organisms, such as the vinegar fly,* Drosophila melanogaster*, are being utilized to provide novel insights into the molecular mechanisms involved. In this review, we highlight recent advances in modelling tumorigenesis using the* Drosophila* model, focusing on the cooperation of oncogenes or tumour suppressors, and the interaction of mutant cells with the surrounding tissue in epithelial tumour initiation and progression.

## 1. Introduction:* Drosophila* as a Model for Understanding Human Cancer

For over 100 years, research utilizing the powerful genetics of the vinegar fly,* Drosophila melanogaster*, has contributed to the understanding of fundamental cellular and developmental processes relevant to the medical field (reviewed in [1, 2]). Indeed, research using the* Drosophila* model had now been granted five Nobel Prizes for Medicine or Physiology. Moreover, the* Drosophila* model has proven to be a highly suitable system for understanding cancer and in developing cancer therapies (reviewed in [3–15]). Use of* Drosophila* as a model organism for cancer research was pioneered by genetic screens, conducted in the late 1900s, which identified many* Drosophila* tumour-causing mutations (reviewed in [16, 17]). Many of these were novel tumour-suppressor genes or oncogenes, which were subsequently shown to also have tumourigenic properties in mammalian systems and to be involved in human cancer (reviewed in [8, 9, 11, 18, 19]).

The strengths of the* Drosophila* model for cancer research lie in the evolutionary conservation of genes and signalling pathways between flies and humans, its lower genetic redundancy, simpler biology, rapid life cycle, and powerful genetics (reviewed in [1, 2, 15]). Due to the sophisticated genetic tools available, cancer-causing mutations can be studied in a tissue-specific or mosaic context. In the study of tumorigenesis in* Drosophila*, the developing epithelial tissues of the* Drosophila* larval imaginal discs that generate the adult eye-antenna or wing-thorax or the epitheliums of the adult intestine are commonly used (reviewed in [7, 20–22]). Indeed, it is mosaic (clonal) analyses using these epithelial tissues that have enabled new insights into the initiation and progression of cancer. In this review, we highlight recent studies focusing primarily on* Drosophila* epithelial tissues, showing how cooperating interactions between cells, and between mutations in oncogenes or tumour-suppressor genes, drive cancer initiation and progression.

## 2. Cell Competition and Cooperating Interactions between Cells in Tumorigenesis

**Figure 1:**
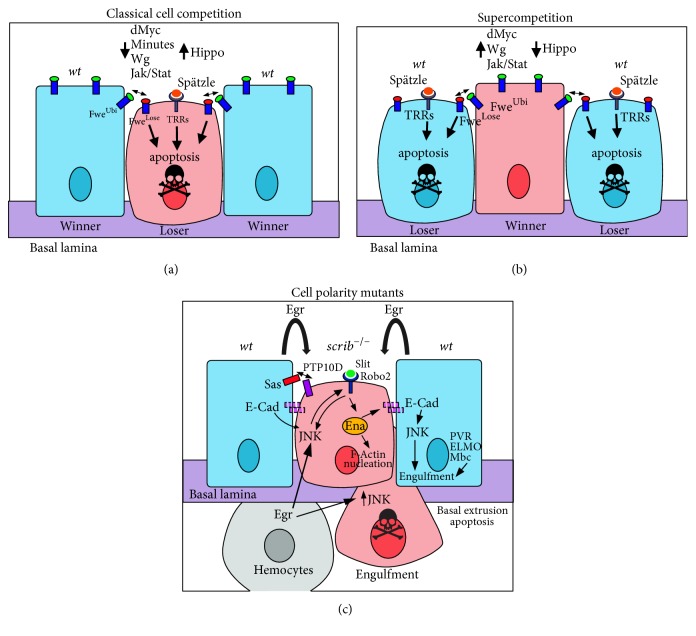
*Cell competition mechanisms*. The three main types of cell competition are shown. Mutant cells are in pink,* wild-type* cells are in blue, hemocytes are in grey, and the basement membrane (basal lamina) is in purple. (a) Classical cell competition: within an epithelium, cells with reduced levels of dMyc, ribosomal subunits mutants (minutes), Jak-Stat or Wg signalling, or high levels of Hippo signalling (losers) are eliminated by apoptosis, induced by the surrounding* wild-type* cells (winners). The loser cells express on their cell surface the Flower-Lose (Fwe^Lose^) isoform (red dots), which marks them for elimination when in contact with the surrounding* wild-type* cells that express the Flower-Ubi (Fwe^Ubi^) isoform (green dots). Additionally, signalling via the Spätzle ligand and Toll-Like Receptors (TLRs) in the loser cells triggers cell death via upregulation of cell death inducers, Rpr or Hid. Cells with upregulated Hippo signalling (or* yki *mutants) exhibit decreased dMyc levels, but cells with decreased ribosomal function, Jak-Stat, or Wg signalling undergo dMyc-independent cell competition. (b) Supercompetition: cells with high levels of dMyc, Jak-Stat, increased Wg signalling, or decreased Hippo signalling show “supercompetitor” behaviour and induce apoptosis in neighbouring* wild-type* cells. This occurs via the Flower-code or via Spätzle-TLR signalling in the loser cells. (c) Cell polarity mutant cell competition: cell polarity-impaired mutant cells are recognized by their epithelial neighbours or hemocytes (grey) and the TNFR-JNK signalling ligand, Egr (TNF), which is secreted by the* wild-type* epithelial cells or hemocytes. Mutant cells are removed by JNK-dependent and caspase-dependent apoptosis. JNK activation in neighbouring* wild-type* cells together with PVR, ELMO, and Mbc signalling is required in the* wild-type* cells for the removal of the dying cells. Hemocytes play the predominant role in engulfment and removal of the dead cells. The interaction of PTP10D in the mutant cell with SAS in the* wild-type* cell is important for “loser” cell fate of the polarity-impaired mutant cell. The Slit-Robo-Ena signalling pathway plays an important role in basal extrusion of the mutant cell, where the hemocytes are localized.

Epithelial tumours can be initiated by multiple molecular lesions, including deregulation of signalling pathways and the perturbation of cell polarity/morphology, such as those generated by loss of function of the cell polarity regulator, Scribbled (Scrib) [15, 23–25]. The clonal-analysis approach has enabled the molecular interactions between the developing epithelial tumour and the surrounding normal tissue, the innate immune system, or distant organs to be revealed (reviewed in [6, 26–30]). The interaction between a tumour cell and the surrounding normal cells in an epithelium is important in determining whether the tumour cell survives and proliferates or is eliminated. The phenomenon of “cell competition,” a surveillance mechanism that compares the fitness of cells in an epithelium, is critical for the active elimination of cells of lower fitness (losers) by cells of greater fitness (winners) within an epithelial tissue (reviewed in [29, 31–33]) (Figure 1). Cell competition involves the interaction of cells and cell-surface molecules or a modified innate immune signalling pathway, leading to caspase-mediated apoptosis of the loser cells by the winner cells. The mechanism of cell competition depends upon the molecular lesion. Cells with low levels of the cell growth regulator, dMyc, or of ribosomal proteins, which reduce cellular growth, are recognized and eliminated differently from those where cell polarity is impaired [34–39] (Figure 1(a)). Differentially expressed cell-surface receptor isoforms of the Flower protein [37, 38] or modified innate immune signalling involving Toll-Like Receptor-Nf*κ*B (TLR-Nf*κ*B) signalling are involved in the elimination of low dMyc or ribosomal protein expressing cells [35].

Clonal alterations in signalling pathways such as Wingless (Wg/Wnt), Jak-Stat, and the Hippo negative tissue-growth control pathways can also induce cell competition (reviewed in [33, 36, 40]). Impairment of Hippo signalling, in addition to upregulating cell cycle and cell survival genes, leads to the upregulation of dMyc and results in a supercompetitor phenotype, where the surrounding* wild-type* cells are actively eliminated [41, 42] (Figure 1(b)). However, the cell competition mechanism that occurs upon differences in the Wg or Jak-Stat signalling occur by dMyc-independent mechanisms, which are currently not well defined [43, 44].

By contrast,* scrib* mutant cell competition requires the interaction of a membrane tyrosine phosphatase, PTP10D, on the loser cell and a membrane protein, Sas, on the winner cell, which results in repression of Epidermal Growth Factor Receptor- (EGFR-) Ras small-GTPase signalling and the activation of the Jun N-terminal Kinase (JNK) signalling in the loser cell [34] (Figure 1(c)). Additionally, JNK signalling activates the Slit-Robo-Ena signalling pathway leading to downregulation of E-Cadherin (E-Cad) and the basal extrusion of* scrib* mutant cells, where they die [45, 46]. Indeed, downregulation of E-Cad appears to be important in* scrib* mutant cell extrusion and elimination, since overexpression of E-Cad in* scrib* mutant clones reduced cell extrusion and promoted clonal overgrowth [45]. JNK signalling also overrides the impaired Hippo signalling in* scrib* mutant cells in a clonal context, preventing their overgrowth [47, 48]. Furthermore, differing levels of dMyc or Jak-Stat signalling between the polarity-impaired mutant cells and the surrounding* wild-type* cells has also been implicated in the elimination of the mutant cells in particular contexts [49–51].

**Figure 2:**
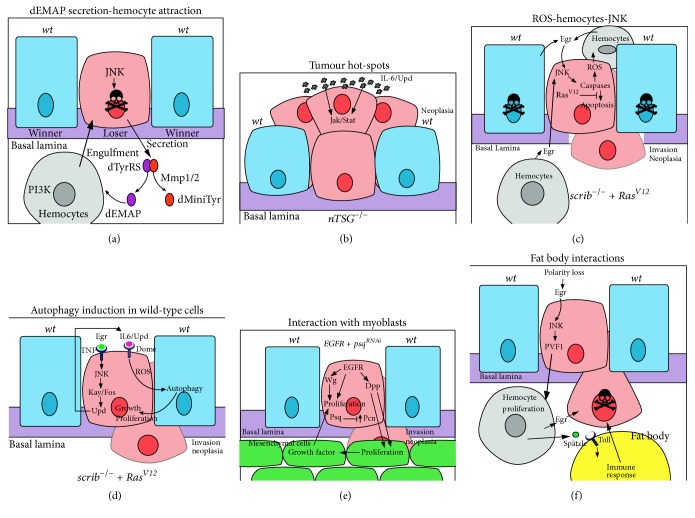
*Cooperative interactions between the tumour and surrounding cells in tumorigenesis*. Interactions between cells are shown that result in either the death of the mutant cell or cell survival, proliferation, and neoplastic transformation. Mutant cells are in pink,* wild-type* cells are in blue, hemocytes are in grey, myoblasts (mesenchymal cells) are in green, a fat body adipocyte is in yellow, and the basement membrane (basal lamina) is in purple. (a) dEMAP secretion-hemocyte attraction: JNK signalling in a cell polarity-impaired loser cell transcriptionally upregulates MMP1, which acts to cleave secreted dTyrRS to form dEMAP and dminiTyr. dEMAP attracts hemocytes to the loser cell by upregulating PI3K signalling in the hemocytes, which is required for chemotaxis and possibly engulfment of the loser cell. (b) Tumour hot-spots: neoplastic tumour-suppressor mutants (nTSGs) induce tumours more preferably, in regions where there is a stiff basal lamina and there are developmentally high levels of the Upd (IL-6) ligand to elevate Jak-Stat signalling, which promotes cell survival and proliferation of the tumour cells. (c) ROS-hemocytes-JNK: in* scrib* mutant* Ras*^*V12*^-expressing tumour cells, a feedback loop between the hemocytes and the mutant cells promotes tumorigenesis. In the mutant cells, Ras signalling and caspase activation leads to ROS production that is released from the cells and promotes hemocytes to produce Egr (TNF). Egr signals via the TNFR-JNK pathway in the mutant cell leading to the upregulation of caspase activity, and some apoptosis, which is required for tumour overgrowth and invasion. Due to the disruption of the peripodial epithelium in large* scrib* mutant* Ras*^*V12*^-expressing tumours, hemocytes most likely interact with the tumour on both apical and basal sides. (d) Induction of autophagy in surrounding* wild-type* cells:* scrib* mutant* Ras*^*V12*^-expressing tumour cells are metabolically stressed, which leads to ROS production. Egr-JNK signalling leads to the transcriptional upregulation of Upd, ligands for the Dome-Jak-Stat signalling pathway, which is elevated in the mutant cells. Jak-Stat signalling and ROS production are required for the induction of autophagy in the surrounding* wild-type* cells, and also at distant sites, such as the fat body, muscle, and gut (not shown), which facilitates tumour growth and neoplastic transformation, possibly through supplying amino acids, glucose, and other nutrients to the tumour cells. (e) Interactions with myoblasts: in* EGFR*-overexpressing* psq*-knockdown tumours cooperative interactions are observed between the tumour cells and the surrounding myoblasts (mesenchymal cells). EGFR induces Wg and Dpp expression, and* psq* knockdown leads to increased levels of the extracellular matrix protein, Perlecan (Pcn). Wg acts to promote proliferation of the tumour cells, whilst Dpp, facilitated by Pcn in the basal lamina, stimulates proliferation of the myoblast cells. In turn, the myoblast cells provide unidentified growth factors that drive proliferation and neoplastic transformation of the tumour cells. Myoblasts also supply Egr (not shown), which would be expected to activate the TNFR-JNK signalling pathway in the tumour cells. (f) Interactions with the fat body: polarity-impaired tumours through Egr-JNK signalling upregulate PVF1, a ligand for the PVR receptor on hemocytes, which promotes hemocyte proliferation. Hemocytes, in turn, supply Egr to the tumour cells, and the Toll Receptor ligand, Spätzle, to the fat body, which induces innate immune system signalling in the fat body. These interactions are required to induce apoptosis of tumour cells.

In addition to cell competition, the interactions between the tumour and its microenvironment are critical for whether the tumour cells will undergo apoptosis or overgrow and eliminate the normal tissue (Figures 1(c) and 2). Interactions between the surrounding* wild-type* epithelial cells, mesenchymal cells (myoblasts), or macrophage-like innate immune system cells (hemocytes) contribute to the fate of the tumour cells [52–62]. Mechanistically, the emerging picture from the study of neoplastic tumours generated in imaginal epithelial tissues (such as with mutants in the neoplastic tumour-suppressor gene (nTSG),* scrib*), is that tumour development occurs through the cooperative interaction of factors produced from surrounding epithelial cells or hemocytes and feed-forward mechanisms within the tumour cell amplifying this loop (Figure 2). Hemocytes are attracted to sites of cell competition by the secretion of fragments of the Tyrosyl-tRNA synthetase protein (dminiTyr and dEMAP), which is triggered by JNK activation and Metalloproteinase (MMP) dependent cleavage in dying loser cells [63] (Figure 2(a)). Mechanistically, dEMAP upregulates PI3K signalling in the hemocytes, which is required for hemocyte chemotaxis [64] and may be important in engulfment of the dying cells [63].

A highly important pathway in cell-cell interactions that triggers tumour cell death is the Tumour Necrosis Factor (TNF), Eiger (Egr), pathway (Figure 1(c)). Egr signals via the TNF receptor (TNFR), Grindenwald (Grnd), and leads to the activation of the JNK signalling pathway in the tumour cell, which, through the activation of caspases, results in caspase-mediated apoptosis of initiating tumour cells [143]. Egr can be produced from the adjacent* wild-type* epithelial cells, myoblasts, or the hemocytes [52, 55, 61, 89, 144]. The* wild-type* cells on the border of the mutant clone also require JNK signalling, though in a nonapoptotic role, and the induction of PVR (PDGF/VEGF receptor homolog)-ELMO (Ced-12 homolog)-Mbc (Dock180 homolog) signalling to induce engulfment of the mutant cells [54] (Figure 1(c)). Whilst there is evidence that* wild-type* epithelial cells engulf the* scrib* mutant dying cells [54], hemocytes play the predominant role in this process, as well as in cell competition due to variations in dMyc or ribosomal protein levels [145, 146]. Furthermore, in tumour development, microenvironmental “hot-spots” have been revealed where the tumour has a greater chance of progressing, which has parallels with mammalian systems [27, 147]. Molecularly, the “hot-spots” are due to endogenously higher levels of Jak-Stat signalling and the presence of a stiff basement membrane extracellular matrix, resulting in extrusion of the tumour cells apically, where they survive (Figure 2(b)). Conversely, in “cold-spots,” tumour cells extrude basally from the epithelium and die, perhaps due to exposure to hemocytes (see below). Molecularly, the level of Slit-Robo-Ena signalling is important for the direction of cell extrusion and therefore dictates whether the aberrant cells will be eliminated by basal extrusion, remain in the epithelium and overgrow, or are apically extruded into the lumen and progress to invasive tumours [45, 46].

By contrast, if cell death is prevented in the mutant cells by blocking caspase activity or upregulation of a cell survival pathway, such as the EGFR-Ras signalling pathway, then the cells survive and form invasive tumours [23, 65, 66, 89–91, 144]. This occurs since TNFR-JNK signalling is repurposed to promote cell morphology changes and migratory cell behaviour (reviewed in [143]). Ras signalling prevents caspase-mediated cell death, and instead caspase activity induces the formation of reactive oxygen species (ROS) within the cell and promotes their secretion [57] (Figure 2(c)). Extracellular ROS, in turn, attracts hemocytes, which secrete TNF and amplify the JNK signalling pathway in the tumour cell [57]. Interestingly, a recent report revealed that ROS, released from the* scrib* mutant* Ras*^*V12*^-expressing tumour cells, promotes autophagy (a catabolic process that degrades cellular macromolecules and organelles to provide energy) in the surrounding* wild-type* cells, as well as systemically in gut, muscle, and adipose tissues [60] (Figure 2(d)). The induction of autophagy may serve to provide glucose, amino acids, and other nutrients that facilitate tumour growth. In the* scrib* mutant* Ras*^*V12*^*-*expressing cells, Egr-JNK-Fos (Kay) signalling together with Ras-MAPK signalling generates metabolic stress, leading to ROS production [60, 101]. JNK and impaired Hippo signalling in these tumour cells also result in the transcription of* unpaired 1–3 (upd1*–*3)*, which encode IL-6-related ligands for the Domeless (Dome) receptor of the Jak-Stat pathway, thereby activating this signalling pathway and promoting tumour growth [148]. Interestingly, Upd1–3 acts in an autocrine manner in the tumour cells to promote autophagy in the neighbouring* wild-type* cells, most likely by stimulating ROS production or secretion [60].

Furthermore, myoblast cells are thought to provide growth factors, which are currently unidentified, to the epithelial tumour cells to stimulate proliferation and survival [52] (Figure 2(e)). In an* EGFR*-driven* pipsqueak* knockdown neoplastic tumour model, EGFR signalling induces upregulation of Wg, which promotes epithelial tumour cell proliferation, but tumour growth is dependent on the neighbouring myoblast cells. Interestingly, a codependency occurs between the epithelial neoplastic tumour cells and the mesenchymal cells, whereby the TGF*β*/Bone Morphogenetic Protein- (BMP-) family morphogen, Decapentaplegic (Dpp), produced in the epithelial cells promotes the expansion of mesenchymal cell compartment, and, in turn, the myoblast cells are required for epithelial cell tumorigenesis [52]. Recent studies have shown that the myoblast cells also produce Egr [61], which, via TNFR signalling, promotes tumorigenesis when cell death is blocked in the epithelial tumour cells. Despite studies showing the importance of Egr in inducing JNK signalling in neoplastic tumours [89], an intrinsic mechanism also exists to elevate JNK signalling in the tumour cells, involving Rho1-GTPase signalling and activation of the JNKKK, Wallenda [61, 149]. Thus, initially, impairment of cell polarity may trigger JNK activation through the Rho1-Wallenda pathway, and, subsequently, myoblasts and hemocytes in the tumour microenvironment are stimulated to produce Egr, thereby amplifying JNK activation in the tumour.

In addition to interactions between the epithelial tumour cells and their local microenvironment, there is also evidence for communication between the hemocyte and the fat body adipocytes [56] (Figure 2(f)). In polarity-impaired neoplastic tumour-bearing larvae, hemocytes supply the Toll ligand, Spätzle, to the fat body adipocytes, which leads to induction of the Toll-NF*κ*B innate immune response signalling pathway in the adipocytes and the production of immune peptides. Egr-JNK signalling in the tumour cells also contributes to the cellular crosstalk, since it results in the transcriptional upregulation of the ligand, PVF1, which, through the PVR signalling pathway, stimulates hemocyte proliferation, thereby elevating Spätzle production from the hemocytes and innate immune signalling in the fat body. This mechanism is required to restrain tumour growth, since knockdown of Spätzle expression in the hemocytes results in reduced Toll pathway signalling in the fat body and reduced tumour cell death. However, whether the fat body-induced immune response only functions to activate the hemocytes, or also by secretion of diffusible signals, to promote tumour cell death, is presently unclear. Moreover, since a Spätzle-modified Toll signalling pathway leading to caspase activation has been observed in dMyc and ribosomal protein cell competition mechanisms [35], the hemocytes might also supply Spätzle to the tumour cells to contribute to their death. Consistent with this, crosstalk between the Toll and JNK signalling pathways in triggering cell death occurs in eye-antennal and wing epithelial tissues [150]. In these tissues, JNK signalling in the epithelial cells induces Spätzle upregulation in the surrounding peripodial membrane cells by an unknown mechanism, which, in turn, activates Toll-Nf*κ*B signalling in the epithelial cells. Thus, Spätzle production by hemocytes or peripodial membrane cells, together with Egr-JNK signalling and signals from the fat body, may all be involved in triggering tumour cell death.

To summarize, in cell competition within epithelial tissues, signals from the myoblasts, the extracellular matrix, the cellular innate immune system, and systemic responses all influence whether the tumour cells will be eliminated or survive and progress to form overgrown invasive tumours. Moreover, if cell death of the tumour cells is blocked, tumour intrinsic and cell-cell signalling pathways that are normally antitumorigenic can instead become tumour-promoting (see below). Cell competition mechanisms are conserved in mammalian systems (reviewed in [36, 39, 151, 152]), and the tumour microenvironment plays a key role in mammalian tumorigenesis (reviewed in [153–156]). Thus, the findings from these* Drosophila* studies of cellular interactions in tumorigenesis are likely to provide new insights into the understanding of human cancer initiation and progression.

## 3. Cooperation Interactions between Oncogenic or Tumour-Suppressor Mutations in Tumour Initiation and Progression

The development of malignant cancer requires the deregulation of many processes, including increased cell proliferation, reduced differentiation and apoptosis, increased invasion, and altered metabolism (reviewed in [157]). There are only a few tumour-causing genes that when individually knocked down or overexpressed in whole epithelial tissues or large domains, are capable of inducing all the hallmarks of cancer that can be modelled in* Drosophila* (reviewed in [3, 11, 15, 158]). Many genes, when deregulated, can cause hyperplastic tumours, characterized by increased tissue growth that are still capable of differentiating, but only a few result in neoplastic tumours, in which the tissue overgrows and shows reduced differentiation and a loss of tissue architecture (reviewed in [159]). Genes capable of conferring many hallmarks of cancer when knocked down or mutated in large domains in epithelial tissues are the junctional (cell polarity regulators, Scrib, Dlg, and Lgl) and endocytic (such as Rab5) neoplastic tumour suppressors. Moreover, recent studies have shown that* lgl* mutant tumours, in addition to possessing other cancer hallmarks, are able to induce an angiogenesis-like process in* Drosophila*, tracheogenesis, in order to obtain an increased oxygen supply [160, 161]. A gene capable of conferring neoplastic overgrowth when expressed in large epithelial tissue domains is the activated version of the receptor tyrosine kinase gene,* PVR* [159, 162, 163]. Additionally, a recent study has shown that expression of the oncogenic fusion between the KIF5B kinesin motor protein and the Ret tyrosine kinase, KIF5B-Ret, promotes many hallmarks of cancer in tracheal epithelial cells [164]. However, as cancer arises from mutations that occur in single cells surrounded by normal tissue, it is uncommon for perturbations in any one gene to confer all the properties that are required for a normal cell to transform into a proliferative-invasive cancer within the context of a* wild-type* epithelium, since cell competition leads to the elimination of aberrant cells. Even with potent tumour-causing mutations, when generated clonally or by induction in a tissue domain, growth of the tumour beyond a certain size is required to overcome apoptosis induced by cell competition [49, 165]. Thus, the phenomenon of cell competition is one reason why at least two mutations are required for tumour progression when initiated in single cells or small patches of cells, particularly concerning mutants in cell polarity or endocytosis regulators. We will now highlight various cooperative tumorigenesis mechanisms that have been modelled in* Drosophila*, focusing primarily on epithelial tissues (summarized in Table 1), and discuss the important insights these studies have revealed. We will first cover the genes/pathways involved in cell death, caspases (cysteine proteases), and the JNK signalling pathway, since they can have context-dependent roles in tumorigenesis.

### 3.1. Caspases in Cooperative Tumorigenesis: Context Dependency

Blocking cell death in the mutant tissue (via blockage of effector caspase activity by overexpressing p35) can, in some cases, enable the survival of the mutant cells, thereby revealing their tumourigenic properties. Examples of caspases acting in a tumour-suppressor role occur in epithelial tissues containing* scrib, rok, mud, Sin3a, Snr1, Csk,* or* frazzled* mutant cells [125, 141, 166–168] or overexpressing a subunit of the Vacuolar ATPase (V-ATPase) complex, Vha44 [169]. However, caspases can also be oncogenic in some contexts. Indeed, activating certain caspases at low levels, insufficient to induce cell death (at least not rapidly), can promote an invasive phenotype [168, 170]. Similarly, caspase activity within the tumour is also required for growth of tumours generated by mutations of the endocytosis regulator, Rab5 [165]. Caspase activity is also observed in wing epithelial tumours generated by mutation of the cell polarity regulator gene,* lgl*, which correlates with JNK pathway activation and is important for tumour invasion [161]. Additionally, in polarity-impaired* Ras*^*V12*^ epithelial tumours, described above, reducing cell death by knocking down caspase activity reduces tumorigenesis [57]. Thus, caspase activity can be tumour promoting or tumour suppressing, depending on context. These findings have implications for cancer therapy, which is designed to induce caspase-mediated cell death, since mild-to-moderate activation of caspases may instead promote tumour growth and invasive behaviour.

### 3.2. The JNK Signalling Pathway in Cooperative Tumorigenesis: Context Dependency

The JNK signalling pathway can also have context-dependent roles in tumorigenesis in* Drosophila* and in mammalian systems (reviewed in [143, 171–174]). In some types of cell competition, such as that induced by polarity impairment, the JNK pathway is required to promote apoptosis and therefore is inhibitory for tumour progression (acting as a tumour suppressor) [23, 91, 136]. In these cases, when JNK signalling is blocked using a kinase-dead dominant-negative JNK transgene* (bsk*^*DN*^), tumour cells delaminate from the epithelium, overgrow, and invade into the surrounding epithelium. This occurs in clones for cell polarity regulators, such as* scrib* or* lgl* mutants, but also occurs upon overexpression of an activated version of* aPKC* or* wild-type crb* in clones in the developing eye epithelia when* bsk*^*DN*^ is coexpressed [66, 71] (Table 1). The mechanism by which JNK-independent cell invasion occurs in these cases is unknown. Interestingly, in* lgl* mutant clones expressing* bsk*^*DN*^, large GFP-marked tumours are observed in the eye, and clumps of GFP-marked cells occur elsewhere in the head and also in body of the pupae/pharate adult [71]. Cooperative interactions also occur upon blocking JNK and activating other signalling pathways to promote tumorigenesis. Overexpression of the Src tyrosine protein kinase gene,* Src64B*, activates JNK signalling and leads to cell death in the eye-antennal epithelium, but when* bsk*^*DN*^ is coexpressed, tumour overgrowth occurs, in a mechanism involving upregulation of the actin-cytoskeletal regulators, Rac1 and Dia, as well as Ras signalling, which inhibit the Hippo pathway, thereby promoting tumour growth [79]. Similarly, in mutants affecting endocytosis, such as* Vps4*, blocking JNK signalling promotes the formation of neoplastic tumours in epithelial tissues, by an unknown mechanism [175].

In another model of tumorigenesis in the developing eye, mutants in* frazzled* (an ortholog of mammalian Deleted in Colorectal Cancer, DCC, a regulator of axon guidance), combined with the blockage of apoptosis by expression of the effector caspase inhibitor, p35, results in elevated JNK and Rho1 activity and promotes cell invasion [125]. However, photoreceptor differentiation still occurs, leading to the migration of differentiated photoreceptor cells to distant sites. Blockage of JNK signalling in* frazzled* mutant* p35* expressing cells enhances the invasive phenotype in a Rho1-dependent manner (Table 1).

Another tumour type, where blocking JNK promotes an invasive phenotype, is the* eyeful* model [116] (Table 1). In this model, overexpression of the Notch ligand, Delta, combined with overexpression of the transcription factor genes,* lola* and* pipsqueak*, in the developing eye, promotes an invasive phenotype but does not affect differentiation, resulting in differentiated photoreceptor cells located at distant sites. This phenotype is dependent on the Polycomb group chromatin-remodelling factor, histone deacetylases, and reduced expression of Rbf1 (the* Drosophila* ortholog of the retinoblastoma tumour suppressor) [116]. Using this model, another group found that overexpression of* atonal* (a transcription factor gene, involved in eye differentiation) reduces the* eyeful* invasive phenotype, whereas knockdown of* atonal* enhances it [120] (Table 1). Atonal functions by inducing JNK activity and possibly enhances cell death and therefore blocking JNK results in restoration of the invasive phenotype [120]. Mammalian atonal, ATOH1, also acts as a tumour suppressor, which may also involve JNK activation [176]. Consistent with the involvement of JNK as a tumour suppressor in this context, in* Delta*-expressing* Drosophila *eye epithelial cells, blocking JNK activity also enhances the invasive phenotype [120]. How invasion occurs upon blocking JNK activity in* Delta*-expressing cells is unknown. Altogether, these examples indicate that blocking JNK can promote cell survival of tumourous cells and that alternate mechanisms promote cell invasion. In studies where mechanistic insights were obtained, these have indicated the involvement of Rho1 or Rac1, which are known regulators of the actin cytoskeleton in cell migration (reviewed in [177]), and the activation of these small-GTPase may very well be involved in other cases of JNK-independent cell invasion.

In contrast to the above examples that highlight a tumour suppressive role for JNK signalling, in other contexts, the JNK pathway can function as a tumour promoter, by altering cell morphology, driving cell invasion, and blocking differentiation. For example, in* lgl* mutant wing epithelial tissue, JNK activation promotes cell morphology changes that potentiates the loss of apicobasal cell polarity and enables tumour formation [178, 179]. Furthermore, in* scrib, dlg*, or* lgl* mutant* Ras*^*V12*^-expressing clones in the developing eye (see below), inhibition of JNK prevents invasive behaviour of cells into the brain lobes-ventral ganglion and promotes differentiation and pupariation [66, 89–91, 93]. Similarly, in wing epithelial tissues overexpressing the Vha44 component of the V-ATPase, which activates JNK signalling and results in invasive tumours, blocking JNK suppresses the invasive phenotype [169]. Additionally, in eye epithelial tissue activation/overexpression of the Rho1 or Rac1 small GTPases (which regulate actin polymerisation and F-actin/Myosin II contractility) also cooperate with* Ras*^*V12*^ to promote invasive overgrowth, dependent upon increased JNK activity [76, 77] (Table 1). In another model, impairment of the Sds22/PP1 phosphatase in* Ras*^*V12*^-expressing cells in the anterior-posterior boundary of the developing wing epithelium, in a JNK-dependent manner, leads to invasive tumours [96] (Table 1). Here, Myosin II activation is also required for invasion, which mechanistically may involve regulation of the JNK pathway by Rho1-Rok-Myosin II signalling, as has been observed in other contexts [61, 76, 77, 149, 180]. Indeed, JNK's oncogenic role in cooperative tumorigenesis is evident in experiments showing that overexpressing JNK pathway genes in combination with* Ras*^*V12*^ in the developing eye epithelium induces invasive tumour growth [76, 90, 91, 149]. Moreover, overexpression of the E2 ubiquitin ligase, Ben/dUev1a, which activates JNK signalling, also cooperates with* Ras*^*V12*^ to promote invasive tumour growth [94] (Table 1). More recently, loss of function mutations in the PP6 phosphatase have been shown to act upstream of the Tak1 protein kinase, a JNKKK, to induce invasive tumorigenesis in* Ras*^*V12*^-expressing eye-antennal epithelial cells [97]. Furthermore, in the adult* Drosophila* hindgut epithelium, JNK activation through the Egr (TNF) pathway, in response to bacterial infection, also cooperates with* Ras*^*V12*^ to promote invasive overgrowth [92, 98] (Table 1).

In summary, the JNK pathway is an important player in cooperative tumorigenesis but dependent on context it can have a tumour-suppressing or tumour-promoting role. Due to this context dependency, which is also observed in mammalian systems [173, 174], the activation of JNK alone in a tumour is not a clear diagnostic or prognostic marker of outcome, and knowledge of other molecular defects is required to predict tumour behaviour.

### 3.3. Cooperation between Cell Polarity Impairment and Oncogenes

Impairment of cell polarity is a powerful force in tumorigenesis (reviewed in [15, 19, 181]). When cell polarity genes* (scrib, dlg*, and* lgl)* are mutated or knocked down in a clonal context, aberrant mitotic spindle orientation, cell polarity impairment, ectopic cell proliferation, and aberrant differentiation occur, but, despite this, malignant tumours do not form, and the mutant tissue is mostly eliminated by JNK-mediated cell death [23, 90, 91, 136, 141, 182]. However, in arguably the first demonstration of* Drosophila* cooperative tumorigenesis, expression of oncogenic* Ras (Ras*^*V12*^) or* Notch (*Notch^*intra*  (*Act*)^) in* scrib* mutant clones prevents their elimination by cell death and instead promotes cell proliferation to produce overgrown undifferentiated and invasive tumours [23, 65, 90, 91] (Table 1, Figure 2(c)). Similar cooperative tumourigenic interactions were also observed for* dlg* and* lgl* mutants and* Ras*^*V12*^ [65] and also for* lgl* and* Notch*^*Act*^ [72]. In these cooperative interactions,* Ras*^*V12*^ and* Notch*^*Act*^ promote cell survival and proliferation, whilst* scrib* mutation leads to aPKC activation, which results in impairment of the Hippo negative tissue-growth pathway, leading to the activation of the downstream cotranscriptional activator, Yki, and tissue overgrowth [47, 48, 183]. Additionally,* scrib* mutation promotes JNK activation, which blocks differentiation and progression to the pupal stage and leads to an invasive cell phenotype through upregulation of MMP1 (a metalloprotease, involved in degradation of the extracellular matrix), Paxillin (a regulator of integrin signalling), Robo (a guidance receptor), and various actin-cytoskeletal regulators [45, 66, 90, 184]. More recently, global expression analyses of* scrib* mutant tissue [148], and* scrib *mutant* Ras*^*V12*^-expressing or* scrib *mutant* Notch*^*Act*^-expressing epithelial tissues [69, 99, 185–187], has revealed the spectrum of deregulated genes that contribute to cooperative tumorigenesis. In addition to members of the JNK and Hippo pathways, these include Polycomb chromatin-remodelling complex components, the BTB-POZ zinc-finger transcription factor genes,* chinmo* and* fruitless*, the Ets-family transcription factor,* Ets21c*, and the nuclear receptor transcription factor gene,* ftz-F1*. These transcription factors contribute to the switching of the differentiation state of the tissue towards a progenitor cell-like fate, deregulation of signalling pathways, and the promotion of cell proliferation, survival, and invasion. Additionally, genetic screens of* scrib *mutant* Ras*^*V12*^-expressing tumours have revealed the importance of the PI3K signalling pathway [67], and chemical screens have revealed the importance of glutamate utilization enzymes, the TCA cycle, and pyrimidine synthesis [68] for tumour growth.* scrib *mutant* Ras*^*V12*^-expressing tumours, in a JNK-dependent manner, upregulate the diffusible Insulin-like peptide, dILP8 [69]. This, in turn, in the prothoracic gland, leads to the downregulation of the secreted steroid hormone, Ecdysone, which is required for metamorphosis, and therefore pupariation is delayed/prevented, thereby leading to the formation of oversized (giant) larvae [188–190]. In addition,* scrib *mutant* Ras*^*V12*^-expressing tumours secrete the insulin growth factor binding protein, ImpL2, which is an antagonist of Insulin signalling that results in wasting of adipose, muscle, and gonadal tissues in the larvae [191]. Thus, polarity impairment together with the Ras oncogene leads to a plethora of gene expression changes and perturbed signalling pathways, which together promote the tumourigenic phenotype, as well as affecting other tissues in the larvae. Expression profiling and functional analyses of* lgl *mutant epithelial tissue have revealed that, similar to* scrib *mutants, signalling pathways (Hippo and JNK) and cell fate genes are deregulated [72, 160, 178, 179, 192–194]. However, other signalling pathways, such as Notch, PI3K, and Wingless, are also elevated in* lgl* mutant tissue [72, 160, 194–196], but they have not been reported to be so in* scrib* mutant tissue. Therefore, the cooperative tumorigenesis mechanisms of* scrib* and* lgl* mutants with* Ras*^*V12*^ might be slightly different.

Many features of the cooperative tumourigenic interaction between* scrib* mutants and oncogenic Ras are conserved in mammalian epithelial systems, both* in vitro *[197] and* in vivo* in epithelial cells of the mouse prostate, lung, breast, and skin tissue [198–201]. Whilst a complete mechanistic picture is lacking, studies in mammalian cell lines have revealed that Scrib depletion in EGF-stimulated epithelial cells elevates ERK as well as JNK signalling [197], and cell polarity perturbation leads to Hippo pathway impairment [202, 203]. Thus, at least some aspects of the mechanism of cooperation between oncogenic Ras and cell polarity genes mutations have proven to be conserved between* Drosophila* and mammals, and further studies are needed in mammalian systems to reveal whether other downstream events are also conserved. Furthermore,* lgl* mutants cooperate with overexpression of the dMyc transcription factor in the wing epithelium [50], which has also been observed for* scrib* downregulation and Myc in mouse mammary epithelial tissue [204], but whether similar mechanisms are involved is currently not known. Additionally, in the wing epithelial tissue,* lgl* mutant cells that are undergoing cell competition-mediated elimination cooperate with impaired Hippo signalling to generate overgrown neoplastic tumours [49]. However, whether this also occurs in mammalian systems is currently unknown.

Subsequent studies using polarity-impaired epithelial tumour models have revealed novel cooperating genes (see Table 1), which provide insight into mechanisms of tumorigenesis relevant to human cancer. Notable recent examples of these include overexpression of the BTB-POZ transcription factor gene,* abrupt*, which was discovered in a genetic screen to cooperate with* scrib* loss to induce neoplastic tumours in the eye-antennal epithelium [70]. Through target gene identification,* abrupt* overexpression was shown to cooperate with* scrib* mutants in tumorigenesis by downregulation of multiple differentiation genes and deregulation of the Hippo and JNK signalling pathways. Moreover, genes responsive to the steroid hormone, Ecdysone, were downregulated, which contributes to the developmental block at the larval stage, enabling the continuation of invasive tumour growth [70]. Interestingly, the Ecdysone Receptor- (ER-) associated factor, Taiman, which binds to Abrupt in ovarian tissues [205], is required for the growth of* scrib* mutant* abrupt*-overexpressing tumours, and overexpression of* taiman* in* scrib* mutant cells also leads to invasive neoplastic tumours [70]. More recently, Taiman was shown to bind to the Hippo pathway cotranscription factor, Yki, and to control the transcription of a novel set of genes that regulate germ-line stem cell identity [124], although whether these genes are also deregulated in* taiman* or* abrupt*-overexpressing* scrib* mutant tumours has not been investigated.

Another signalling pathway involved in* scrib* mutant tumorigenesis is the Slit-Robo-Ena pathway. This pathway is involved in the basal extrusion of* scrib* mutant tissue from the epithelium, where they die, and downregulation of this pathway results in overgrown (but noninvasive) tumours within the eye-antennal epithelium [45]. Conversely, hyperactivation of the Slit-Robo-Ena pathway in* scrib* mutant or* wild-type* cells results in a hyperextrusive phenotype, with the apically (lumenally) extruded cells forming overgrown tumours, which might occur by the peripodial membrane epithelium preventing access of the innate cellular immune system cells to the tumour [45, 46]. However, it is also possible that the lumenal microenvironment is conducive to tumour cell growth and survival, which may be dependent on morphogens, such as Dpp, produced from the peripodial epithelium [206].

Scribble module genes, but not other apical-basal cell polarity genes, were identified as* Drosophila* neoplastic tumour-suppressor genes; however the downregulation of Crb and Par modules cell polarity genes together with* Ras*^*V12*^ expression in the eye-antennal epithelial also results in neoplastic tumour formation [65]. Furthermore, overexpression of Par1 cell polarity regulator, which inactivates Hippo signalling [74], cooperates with activated Notch signalling in promoting tumourous overgrowth in the eye-antennal epithelium [75], similar to that which occurs with activated* Notch *and* scrib* mutants [23] or* lgl* mutants [72]. It is likely that similar mechanisms are involved in the cooperation of Crb and Par module gene mutants with* Ras*^*V12*^, as well as with* Par1* and* Notch*^*Act*^, as occurs with* scrib* mutants with* Ras*^*V12*^ or* Notch*^*Act*^; however formal demonstration is currently lacking. Interestingly,* canoe* (*cno*,* afadin/AF-6* in mammals), a gene involved in another type of cell polarity, asymmetric cell division [207], has been recently shown to cooperate with* scrib*,* dlg,* or* lgl* depletion in epithelial tumorigenesis [73]. Mechanistically, this synergistic interaction involves the activation of Ras-MAPK signalling, which implicates the* wild-type* function of Cno as well as Scrib, Dlg, and Lgl in the repression of Ras signalling [73], as occurs with the mammalian Cno (Afadin/AF-6) and Scrib [197, 208].

### 3.4. Cooperative Tumorigenesis Involving Actin-Cytoskeletal Regulators

Deregulation of the actin cytoskeleton leads to cell morphology changes, increased tissue growth through impairment of the Hippo pathway, and reduced cell-cell adhesion and can promote invasive phenotypes [76, 77, 177, 209–212]. However, in a clonal context, tissue growth due to deregulated actin-cytoskeletal gene expression is restrained by JNK-mediated cell death, and therefore cell death blockage or oncogenic activation is required for tumorigenesis. Indeed, the activated small GTPases Rho1 and Rac1, which regulate the actin cytoskeleton, cooperate with* Ras*^*V12*^ in tumorigenesis in a JNK-dependent manner [76, 77] (Table 1). Furthermore, downstream of the Rho1-GTPase, the Rok protein kinase, and activated Myosin II, which regulate F-actin filament contractility, cooperates with* Ras*^*V12*^ to promote tumorigenesis [77]. Mechanistically, the contribution of the Rho1-Rok-Myosin II pathway to* Ras*^*V12*^-driven tumorigenesis most likely involves JNK activation [77], and also Hippo pathway impairment, as increased F-actin contractility leads to Yki activation-induced tissue growth (reviewed in [211, 213]). Activation of Rho1, by RhoGEF2 overexpression, also cooperates with overexpression of the Abrupt BTB-POZ transcription factor in inducing neoplastic tumours of the eye-antennal epithelium by blocking expression of differentiation genes [78] (Table 1). It is likely that JNK activation and Hippo impairment are also involved in this cooperative interaction; however this remains to be confirmed.

The Src nonreceptor tyrosine protein kinase, a key regulator of the actin cytoskeleton as well as adherens junctions [214], cooperates with several oncogenes to promote tumorigenesis in* Drosophila *(Table 1). Activation of Src through knockdown of its negative regulator, Csk, together with Ras^V12^ also results in invasive overgrown tumours of the eye-antennal epithelium [80–84]. Mechanistically, Src activates JNK and Stat signalling, modulates the actin cytoskeleton, and impairs Hippo signalling to promote invasive overgrowth in cooperation with Ras^V12^ [79–83, 85]. Moreover, on a high sugar diet, Src-activated Ras^V12^-driven tumours have an altered metabolism and elevate Wg signalling, which leads to upregulation of the Insulin-Receptor gene expression, enabling the tumour cells to become insulin-responsive and aggressively overgrow, whilst other larval tissues are insulin-resistant and hypoplastic [81, 84]. Overexpression of Src64B or Src42A also cooperates with activated Notch signalling to promote tumorigenesis in eye-antennal and wing epithelial tissue, in a mechanism requiring JNK activation in a TNF-independent manner [86]. Given the link between Src and actin-cytoskeletal regulators [85], and the discovery of a mechanism linking Rho1 to JNK activation via the JNKKK, Wallenda [149], a similar mechanism might be involved in the activation of JNK in* Src Notch*^*Act*^ cooperative tumorigenesis. Additionally, Src64B overexpression cooperates with overexpression of the Abrupt BTB-POZ transcription factor in the eye-antennal epithelium by blocking differentiation genes and promoting a progenitor-like cell fate [78]. Although Src expression changes the repertoire of Notch target gene transcription in the* Notch*^*Act*^ tumours [86], whether differentiation blockage is also involved in this tumour type and other Src-driven tumours remains to be determined.

Similarly, when induced in a clonal setting, overexpression of the actin-cytoskeletal regulator, Troponin I, cooperates with* Notch*^*Act*^ expression,* Ras*^*V12*^ expression, and* lgl* mutant* Ras*^*V12*^ expression to promote tumour overgrowth by altering gene transcription [87]. Genes upregulated included those encoding the Insulin Receptor (InR), Rap1 (a Ras-related protein), and Dilp8 (insulin-related peptide), which are likely to affect tumour growth by promoting cell proliferation and in the case of Dilp8 by delaying pupariation through downregulation of Ecdysone production in the prothoracic gland.

### 3.5. Deregulation of Signalling Pathways in Cooperative Tumorigenesis

*EGFR-Ras-MAPK. *The mitogenic EGFR-Ras-MAPK signalling pathway is a powerful inducer of tissue growth but also induces differentiation in* Drosophila* (reviewed in [215, 216]). Moreover, this pathway is important in cancer, as mutations in Ras signalling pathway genes that elevate pathway activity are present in ~30% of human cancers (reviewed in [217–219]). Although oncogenic Ras is a potent inducer of tissue growth, high level of pathway flux leads to senescence or differentiation, thereby limiting tumorigenesis (reviewed in [220, 221]). Thus, additional mutations are required for Ras-driven malignant cancer development.

Oncogenic Ras requires EGFR signalling to potently induce tissue overgrowth in both* Drosophila* and human cells [88]. Mechanistically, this occurs through the endocytosis regulator, Arf6, which is important for the trafficking of the Hedgehog morphogen and activation of the Hedgehog signalling pathway. In* Drosophila*, activated Ras signalling cooperates with many pathways to promote tumorigenesis (Table 1). In addition to mutations/overexpression of cell polarity, actin cytoskeletal, and JNK pathway genes that cooperate with oncogenic Ras in tumorigenesis in* Drosophila* (discussed above), many other cooperative interactions have been revealed in various* Drosophila* epithelial tissues that confer either hyperplastic or neoplastic overgrowth (Table 1). Hyperplastic tumourigenic interactions include the cooperation of* Ras*^*V12*^ with the overexpression of* chinmo* or* fruitless* BTB-POZ domain transcription factor genes [69], and with impaired Hippo pathway signalling [65, 99], which results in enhanced hyperplastic overgrowth of eye-antennal epithelial tissue. In the cooperation of Hippo pathway impairment with Ras activation, a global transcriptome analysis has provided insight into how the differentiation function of Ras signalling is reprogrammed to promote tumorigenesis, by showing that Yki elevates the expression of the Ras target gene,* pointed*, which is crucial for the synergistic tissue growth [99, 222].

Conversely, in the eye-antennal epithelial tissue,* Ras*^*V12*^ cooperates with lysosomal gene loss of function to cause neoplastic overgrowth [100]. Additionally, mutations in the Polycomb complex chromatin-remodelling gene,* polyhomeotic (ph)*, cooperate with* Ras*^*V12*^ in a clonal context to induce eye-antennal tissue neoplastic tumours, which depends on Notch pathway activation [102]. However, loss of* ph* and other Polycomb complex genes, when generated in a whole eye-antennal epithelial tissue, results in neoplastic tumours, which in this context is dependent on ectopic Upd-Jak-Stat signalling [223]. These differences might depend on the level of expression and the region of the tissue affected, but, additionally, in the clonal context, the induction of cell competition might affect the cooperative mechanism involved in neoplastic tumour formation.

Interestingly, autophagy gene knockdown cooperates with* Ras*^*V12*^ to produce different outcomes depending on context [101]. Knockdown of autophagy genes using* UAS-RNAi* lines via the* eyeless-GAL4* driver, or, clonally, within the developing eye epithelium, enhances* Ras*^*V12*^ hyperplastic overgrowth, whereas using the* eyeless-FLP-out Tubulin-GAL4* system, which results in the strong expression of the transgenes throughout the whole eye-antennal epithelium, autophagy gene knockdown together with* Ras*^*V12*^ expression results in neoplastic overgrowth and death at the larval-pupal stage. Mechanistically, the cooperation of* Ras*^*V12*^ with autophagy gene knockdown, in both the hyperplastic and neoplastic tissue overgrowth effects, occurs because oncogenic Ras signalling induces autophagy in imaginal disc epithelial tissues, and consequently the blockage of autophagy at any step of the pathway results in ROS accumulation and activation of JNK signalling [101]. This finding may also be relevant to human cancer, since in human pancreatic cancers, where* K-Ras*^*G*12*V*^ mutations are common, downregulation of several autophagy genes correlates with poor prognosis [101]. Since autophagy inhibitors are being considered for cancer therapy (reviewed in [224]), this study highlights the need for caution with Ras-driven cancers, where inhibiting autophagy might inadvertently exacerbate cancer development.

Overexpression/activation of EGFR also cooperates with several genes in tumorigenesis in* Drosophila* epithelial tissues (Table 1). EGFR cooperates with impaired Hippo pathway signalling, leading to tissue overgrowth [108] and also with the overexpression of the* bantam* micro-RNA (which is a downstream target of Yki [225, 226] and also of EGFR signalling [227]), leading to overgrown invasive tumours [109]. EGFR cooperates with the* bantam* micro-RNA by elevating Jak-Stat signalling due to* bantam* repressing the translation of the Jak-Stat signalling inhibitor, Socs36E. Activated Ras together with knockdown of Socs36E causes similar cooperative effects, showing that, downstream of EGFR, Ras signalling is crucial for this cooperation. Elevated expression of the Snail transcription factor, a driver of the epithelial-to-mesenchymal transition (EMT), also occurred in these tumours, as well as expression of the JNK target, MMP1, suggesting that JNK activation is also involved. This group also discovered that the overexpression of micro-RNAs,* mir-10,* or* mir-375,* cooperates with overexpression of EGFR in promoting invasive overgrown tumours [52]. This cooperation occurs by downregulation of the transcription factor, Pipsqueak (Psq), which leads to increased expression of the extracellular matrix protein, Perlecan (Pcn), resulting in tumour overgrowth by a non-cell-autonomous mechanism involving the surrounding myoblast cells [52, 228] (Figure 2(e), see above). Perlecan promotes Dpp signalling in the myoblasts, supporting their proliferation, and, in turn, the myoblasts provide growth factors that promote epithelial tumorigenesis. More recently, the same group found that overexpression of another micro-RNA and the* miR-200* family member,* miR-8*, cooperates with EGFR overexpression to result in clonal overgrowth, cell polarity loss and invasive phenotypes in the wing epithelial tissue [110]. Curiously, these tumours became polyploid, which was attributed to* miR-8 *repressing the translation of the Septin, Peanut, which is required for cytokinesis. However, although Peanut downregulation was required, it was not sufficient for tumorigenesis with EGFR overexpression, suggesting other* miR-8* targets are also involved. These tumours also acquire a supercompetitor phenotype and are able to induce cell death of, and engulf, their neighbours. In mammalian systems,* miR-200* family downregulation induces an EMT in some settings [229]; however its overexpression occurs in ovarian cancers where cells commonly exhibit polyploidy [230–233]. Thus, this unusual cooperative behaviour, identified in* Drosophila*, might have relevance to certain types of human cancer.

Several studies have also focused on directed modelling in* Drosophila* of EGFR-Ras-driven human cancers, such as lung, colorectal, and glioblastoma cancers (Table 1). In a model of Ras-driven lung cancer,* Ras*^*V12*^ coexpression with* PTEN *knockdown (which elevates PI3K signalling) in the larval-pupal tracheal epithelial cells results in tracheal cell overgrowth and invasive tumours [103]. Colorectal cancer was modelled by knocking down the* adenomatous polyposis coli (apc)* gene and overexpressing* Ras*^*V12*^ in the adult midgut [104, 105], which resulted in hyperplasia. In another study, the adult hindgut was used and* Ras*^*V12*^ was expressed together with* p53, apc, pten *knockdown or* dSmad4*,* apc, *and* pten *knockdown (commonly observed mutations in human colorectal cancers), which resulted in invasive tumours [106]. Glioblastoma was modelled in* Drosophila* by expressing constitutively active forms of EGFR and PI3K, which is commonly observed in human glioblastomas [111, 112, 234]. Genetic analysis revealed that dMyc, Cdc25, Cdk4, and the TORC2 regulatory subunits, Sin1 and Rictor, were important in glial cell tumorigenesis in the brain and eye tissue [111]. Moreover, a genetic screen of the kinome led to the identification of RIOK1 and RIOK2 kinases, which promote mTORC-Akt signalling to drive glial tumour growth [113]. A recent study has also revealed cooperative tumorigenesis in glial tumour growth and invasion between* Ras*^*V12*^ and overexpression of* pico* (a MRL family gene),* chickadee* (*profilin, *encoding an actin-cytoskeletal regulator) or* Mal* (encoding a cofactor of Serum Response Factor (SRF)) [107], suggesting that SRF signalling might be a novel pathway to investigate in human glioblastomas.

*Delta-Notch. *The Delta-Notch signalling pathways play multiple roles in tissue growth and development in* Drosophila*, and ectopic activation leads to overgrowth phenotypes (reviewed in [114, 235, 236]). For neoplastic tumour formation, additional gene mutations are required together with Notch-Delta overexpression/activation, as detailed below. Activated Notch was shown to cooperate with overexpression of the transcription factor Mef2, leading to disruption to the actin cytoskeleton and apicobasal cell polarity [115] (Table 1). This cooperative interaction is JNK dependent, requiring upregulation of Egr [115]. In the* eyeful* model, in which* Delta* is overexpressed with the* psq *and* lola* transcription factor genes [116], cooperative tumorigenesis occurs upon downregulation of the* cut* transcription factor gene, which leads to a disruption to adherens junction-mediated cell-cell adhesion and cell-basement membrane *β*-integrin-mediated adhesion, causing increased invasion [121] (Table 1). In these cooperative interactions of* cut* downregulation with* Delta* overexpression, or with the* eyeful* model, upregulation of the cell death gene,* reaper (rpr), *and elevated PI3K-Akt signalling are involved [121]. The invasive phenotype of these tumours required MMPs, which is a JNK target, but whether JNK was also involved was not determined. Since caspase activation and JNK signalling have been previously linked to invasive cell behaviour in the wing epithelium [168], it is possible that JNK and caspase activation are also involved in the invasive phenotype of* cut* downregulation in the* eyeful* model. Additionally, PI3K or Akt overexpression has been previously shown to cooperate with Delta overexpression in the eye epithelial tissue to induce an overgrown invasive phenotype, which might be relevant to human cancer, particularly T cell acute lymphoblastic leukaemia, where Notch and Akt pathway activation often occurs [117, 237]. The* Delta*-driven invasive phenotype of the* eyeful *model was suppressible by overexpression of the* miR-200* family micro-RNA,* miR-8* [119]. This tumour-suppressor role for* miR-8* is in contrast to its oncogenic role observed in another study [110] and highlights that, like JNK and caspases,* miR-8* also has a context-dependent role in tumorigenesis. Whilst human* miR-200* family micro-RNAs are considered regulators of the epithelial phenotype and tumour suppressors (reviewed in [238]), these discoveries in* Drosophila* highlight that more research is needed to determine whether the* miR-200* family also have context-dependent effects in human cancer.

Mechanistically, in the* eyeful* model,* miR-8* blocks the invasive phenotype by repressing the translation of the Notch ligand, Serrate, and the zinc-finger transcription factor, Zfh1 (an ortholog of mammalian ZEB1, which is an EMT inducer), and, consistent with this, coexpression of* Delta,* or* Serrate,* with* Zfh1* cooperatively promotes an invasive phenotype [119]. This mechanism might be important in mammalian cancer, since* JAGGED1*  (mammalian ortholog of* Delta/Serrate*) is regulated by the* miR-8* orthologs,* miR-200c,* and* miR-141, *in colorectal cancer cell lines [119], and reduced* miR-200* expression is associated with coupregulation of JAGGED1 and ZEB1 proteins in pancreatic and basal-type breast cancer cell lines [239].

The same group also found that another micro-RNA,* mir-7*, when overexpressed, enhances* Delta*-driven tumour overgrowth and promotes invasion in the eye-antennal epithelium, although the cells were still capable of differentiating [118] (Table 1). In this case, the cooperation occurred via blocking Hedgehog pathway signalling, which normally acts to restrict Delta/Serrate-Notch signalling during eye development.* mir-7 *reduced translation of the Hedgehog receptor mRNA,* ihog (interference hedgehog)*, whereas Notch signalling blocked transcription of the coreceptor gene,* boi (brother of ihog)*, thereby leading to reduced Hedgehog signalling and enhancing Delta-Notch-driven tumour growth and invasion. Consistent with the mechanism, blocking Hedgehog signalling by knocking down expression of the Hh pathway transcription factor, Ci, also cooperated with* Delta* overexpression to phenocopy the effect of overexpression of* mir-7* and* Delta* [118]. These studies may provide insights into some forms of human cancer, where the* mir-7* ortholog is overexpressed and oncogenic, such as lung and skin cancers [240], or the Ihog orthologs (BOC and CDO) are downregulated or have a tumour-suppressor functions, such as in pancreatic cancer [241] or rhabdomyosarcoma [242].

*Hippo. *The Hippo tissue-growth control pathway consists of a protein kinase cascade involving Hippo and Warts protein kinases, which when activated, leads to the Warts-mediated phosphorylation and inactivation of the Yki cotranscriptional activator, thereby limiting tissue growth (reviewed in [243]). Hippo is regulated by multiple upstream inputs, including signalling pathways, cell polarity, and mechanical cues (reviewed in [122, 183, 244, 245]). Due to its powerful effect in controlling tissue growth, downregulation of the Hippo pathway is commonly observed in* Drosophila* cooperative tumorigenesis, as well as in human cancers (reviewed in [18, 122, 245, 246]). In addition, to the examples described above, which reveal the cooperation of Hippo pathway impairment in cooperative tumorigenesis with cell polarity impairment and oncogenic Ras, Yki overexpression has also been shown to cooperate with the knockdown of the Brahma (Brm) chromatin-remodelling complex [123] and overexpression of the Taiman transcription regulator [124] (Table 1). Impairment of the Brm-BAP chromatin-remodelling complex (using* brm, snr1,* or* osa* mutants) in epithelial tissues promotes cell cycle entry, alters Ras, Notch, and Dpp signalling, and deregulates Ecdysone responsive genes [247–254]. Brm complex knockdown also deregulates the Hippo pathway in epithelial tissues [255, 256], and therefore it is perhaps surprising that Brm downregulation cooperates with Yki overexpression [123]. Cooperation might occur, due to Brm complex knockdown downregulating the Ras signalling pathway, which decreases cell proliferation and survival [251, 254], and since Yki overexpression provides a strong cell proliferation and survival signal, it would be expected to override decreased cell survival exhibited by Brm complex knockdown alone. However, the recent study showed that Brm-BAP complex depletion, together with Yki overexpression, results in upregulation of Dpp and Wg morphogens leading to neoplastic tumour overgrowth in the larval wing epithelial tissue [123]. Cooperation with Yki and Taiman overexpression occurs by a unique mechanism involving the ectopic expression of germ-line stem cell genes in wing epithelial tissue [124]. This occurs because Yki and Taiman can form a complex leading to upregulation of a new spectrum of Yki targets normally not expressed in imaginal disc epithelial tissue, which alters differentiation.

*Mitotic Checkpoints, Chromosome Instability and DNA Damage Repair Genes. *Genes important in mitotic checkpoints, DNA repair, and genomic integrity play important tumour-suppressor functions in preventing cancer (reviewed in [157]). Indeed, knockdown of the spindle-assembly checkpoint (SAC) gene,* bub3*, which leads to chromosome instability (CIN) and aneuploidy, results in neoplastic tumorigenesis in the wing epithelial tissue when cell death is blocked [127, 140] (Table 1). The results from one group suggested that the mechanism by which this occurs is a SAC-independent function of Bub3 [127], but the second study revealed a novel mechanism that was induced by aneuploidy and cell delamination [140] (see below). A role for the DNA damage checkpoint and DNA repair after exposure to ionizing radiation (IR) has also been revealed in cooperative tumorigenesis [128] (Table 1). Here, IR together with apoptosis inhibition results in overgrowth and cell delamination/migration in the wing epithelial tissue, which is enhanced by knockdown of the DNA repair genes,* okra (DmRAD54)* or* spnA (DmRAD51)*, which are involved in homologous recombination of DNA double-strand breaks, as well as by knockdown of the DNA damage checkpoint genes,* grp (chk1)* and* mei-41 (ATR)*.

An unusual example of cooperative tumorigenesis concerns the Nek2 (NimA related kinase 2) centrosome kinase, which is also involved in the SAC, and its loss of function leads to CIN (reviewed in [257]). However, overexpression of Nek2, which is not expected to cause CIN, cooperates with oncogenic pathways to drive neoplastic tumour formation without apparent effects on CIN [126] (Table 1). Here, expression of the activated form of the Ret tyrosine kinase (*Ret*^*MEN*2*B*^), which mimics oncogenic mutations in human thyroid cancer, and* Ras*^*V12*^ together with* Nek2* overexpression, leads to invasive overgrowth of the eye-antennal epithelial tissue. Similar cooperativity occurs with mutant* csk*, together with* Ras*^*V12*^ and* Nek2* overexpression. The Ret oncogene results in increased signalling through the Ras-MAPK, PI3K, Src, and JNK pathways [258–260]. Nek2 overexpression results in increased Wg signalling and altered expression of Rho1, Rac1, and E-Cadherin, leading to altered cell morphology [126]. Coexpression of Nek2 and oncogenic Ret lead to enhanced local invasion and distant metastases. Mechanistically, Nek and Ret result in elevated expression of MMP1, which is expected to promote extracellular matrix degradation. Additionally, Nek and Ret lead to elevated expression of Diap1 (an antiapoptotic protein, and target of Hippo and Jak-Stat signalling), as well as Wg expression and PI3K signalling, which together are expected to drive tumour growth. A similar cooperative invasive phenotype was observed with elevated Src activity (*csk* mutant) with* Ras*^*V12*^ [126]. PI3K signalling was critical for the cooperative invasive phenotype, since inhibiting PI3K suppressed the cooperative behaviour. Although Nek2 is thought to have a tumour-suppressor function due to its role in the SAC and chromosome stability, it is also oncogenic and drugs are being developed to inhibit its function in cancer therapy (reviewed in [257]). Thus, this study in* Drosophila* provides insight into how Nek2 alone, as well as when combined with oncogenic mutations, promotes invasive properties [126], which is relevant to the understanding of Nek2-overexpressing human cancers.

In summary, the above examples of cooperative tumorigenesis in* Drosophila* tissues, and the delineation of the mechanisms involved, provide insights towards the understanding of various human cancers where these pathways are deregulated and present possible novel avenues for therapeutic intervention.

## 4. The Effect of the Tumour on Normal Tissue Growth and Intertumoural Cooperation

**Figure 3:**
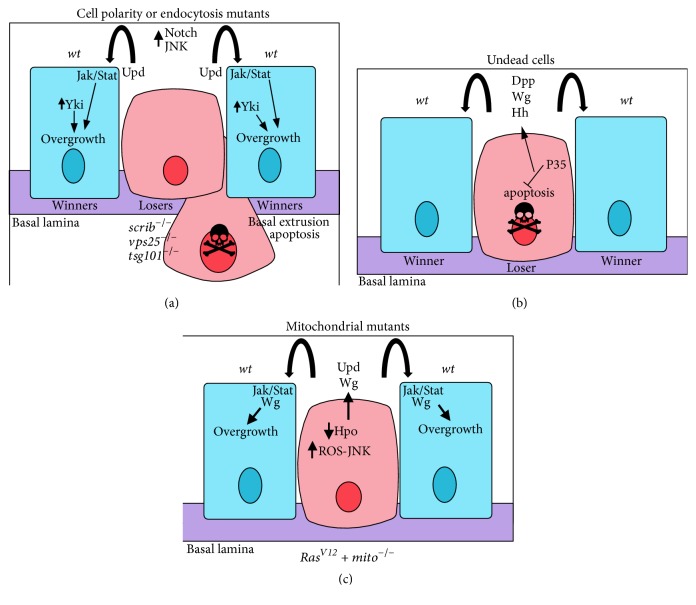
*Non-cell-autonomous overgrowth.* Examples of different types of non-cell-autonomous overgrowth. Mutant cells are in pink,* wild-type* cells are in blue, hemocytes are in grey, and the basement membrane (basal lamina) is in purple. (a) Cell polarity or endocytosis mutant cells are induced by JNK signalling to undergo cell death and induce non-cell-autonomous overgrowth of the surrounding* wild-type* cells. In* vps25* or* tsg101 (ept)* endocytic mutants, which also show apicobasal cell polarity defects, ectopic activation of Notch signalling leads to the expression and secretion of the Dome-Jak-Stat pathway ligand, Upd, which promotes non-cell-autonomous proliferation and overgrowth of surrounding tissue. In* scrib* mutant cells, elevated JNK signalling, and impaired Hippo signalling, leads to transcriptional upregulation of Upd, which activates Dom-Jak-Stat signalling in the surrounding* wild-type* cells, thereby inducing their proliferation. (b) Undead cells, where apoptosis is initiated, but effector caspase activity is blocked, emit morphogens (such as Wg, Dpp, and Hh) that promote proliferation of their* wild-type* epithelial neighbours, thereby leading to non-cell-autonomous overgrowth. (c) Mitochondrial mutants expressing* Ras*^*V12*^ lead to non-cell-autonomous overgrowth. The mitochondrial impairment results in the production of ROS, which induces JNK activation, which, in turn, results in Hippo pathway impairment, leading to expression of the Yki targets, Upd and Wg. Upd elevates Jak/Stat signalling and Wg induces Wg pathway signalling in the surrounding* wild-type* cells to promote their overgrowth.

Not only does the mutant cell depend on the surrounding microenvironment for its proliferation and neoplastic transformation, but there are also examples in* Drosophila* where the tumour induces overgrowth of the genetically* wild-type* surrounding epithelial cells (termed non-cell-autonomous overgrowth) (Figure 3). The sophisticated genetics of* Drosophila* have enabled modelling of complex intertumoural cooperation, through generating genetically different populations of epithelial cells. In mammalian systems, tumours also exert non-cell-autonomous effects on cells in their microenvironment that affect tumour development (reviewed in [153–156]). Additionally, cancer heterogeneity, where different populations of tumour cells interact to promote tumorigenesis, is a recognized phenomenon in mammalian cancer (reviewed in [53, 261–264]). We will now discuss* Drosophila* models of cooperative tumorigenesis, where non-cell-autonomous cell proliferation or tumour heterogeneity occurs.

### 4.1. Non-Cell-Autonomous Cell Proliferation

In clonal settings, the initiation of cell death within mutant tissue results in signalling events that lead to non-cell-autonomous cell proliferation of the surrounding* wild-type* epithelium (reviewed in [28, 33, 265–269]) (Figure 3). In* scrib* mutant clones, non-cell-autonomous proliferative effects on the surrounding* wild-type* epithelial tissue occurs due to JNK-mediated expression of Upd, which in turn induces the Dome-Jak-Stat signalling pathway in surrounding cells [47, 137] (Figure 3(a)). Additionally, there is also evidence that the Hippo pathway is impaired in* wild-type* cells surrounding* scrib* mutant clones [270]. Likewise, clones mutant for the endocytic trafficking genes,* vps25* or* tsg101 (ept)*, also induce proliferation of the surrounding* wild-type* cells, due to impairment of the Hippo pathway and/or upregulation of the Dome-Jak-Stat signalling pathway [47, 137, 270, 271] (Figure 3(a)), which occurs through the aberrant activation of Notch signalling, leading to upregulation of Upd (Dome ligand) in the mutant cells [271, 272].* vps25* mutant cells also lead to Hippo pathway impairment in the surrounding* wild-type* cells [270, 271], which may partially involve signalling through the Fat atypical cadherin [270]. Ectopic Notch signalling alone also leads to non-cell-autonomous tissue growth, as well as cell-autonomous proliferation [271, 273]. Similarly, activation of the Hh pathway in clones, in a Notch-dependent manner, also results in non-cell-autonomous cell proliferation and expression of the Diap1 cell death inhibitor in the surrounding* wild-type* cells [274, 275]. Since* Diap1* is a transcriptional target of Stat and Yki [276, 277], Jak-Stat or Hippo pathway deregulation may be involved. However, in all these examples, cell proliferation is limited. By contrast, in settings where cell death of the mutant cells is blocked by decreasing or preventing caspase activity, substantial overgrowth of the* wild-type* tissue occurs (Table 2). For example, elevated Hh signalling in clones blocked for cell death induces the Dpp morphogen production in the mutant cells, which activates the Dpp signalling pathway in the surrounding* wild-type* cells to induce non-cell-autonomous tissue growth [135]. In this setting, Yki was also upregulated non-cell-autonomously, and together with the Dpp signalling-activated transcription factor, Mad (Smad), induces expression of the* bantam* micro-RNA to promote non-cell-autonomous tissue growth [135] (Table 2). Another example of non-cell-autonomous tissue overgrowth occurs with “undead cells” [267–269]. Undead cells are generated when cell death is initiated by upregulation of an apoptosis regulator (such as Hid or Rpr), but apoptosis is blocked by expression of the p35 effector caspase inhibitor [59, 129–134] (Figure 3(b)). The undead cells continually express and secrete the morphogens, Wg, Dpp, or Hh, which act to elevate these signalling pathways in the surrounding* wild-type* cells, thereby inducing their uncontrolled proliferation. The induction of non-cell-autonomous tissue overgrowth by undead cells is dependent on the activation of JNK signalling in the undead cells, which transcriptionally upregulates the expression of the morphogen genes [129, 268] (Figure 3(b), Table 2). Consistent with this, strong activation of JNK together with Raf (protein kinase that functions downstream of Ras signalling) results in non-cell-autonomous overgrowth of surrounding* wild-type* cells [136]. Additionally, overexpression of* Ras*^*V12*^ together with the actin-cytoskeletal genes,* RhoGEF2*,* Rac1*, or activated alleles of* Rho1 (Rho*1^*V*14^), can initially induce varying degrees of non-cell-autonomous tissue growth; however tumour growth predominates over time [76, 77]. Similar effects are also observed with overexpression of the* abrupt* transcription factor gene with* RhoGEF2* or* Src64B* [78]. Conversely, overexpression of* abrupt* with* Rac1* leads to a strong non-cell-autonomous tissue overgrowth [78]. The mechanism by which this non-cell-autonomous overgrowth is induced is currently unknown but may involve the cells acquiring an undead-like state.

A different form of non-cell-autonomous cell proliferation occurs without cell death of the mutant cells but instead results in the cells acquiring a senescent secretory phenotype [138, 139] (Figure 3(c); Table 2). This new mechanism was discovered in a genetic screen for mutations that cooperate with* Ras*^*V12*^ in the developing eye, which revealed that mutations in mitochondrial oxidative phosphorylation genes together with* Ras*^*V12*^ lead to non-cell-autonomous overgrowth of the surrounding* wild-type* tissue. Mechanistically, this involves the generation of reactive oxygen species (ROS) by the mitochondrial-impaired cells, which then lead to JNK pathway activation. In turn, JNK activation results in impairment of Hippo pathway signalling, and, consequently, elevated Yki induces expression of Upd and Wg, which, respectively, induce signalling through the Dome-Jak-Stat and Wg signalling pathways in surrounding* wild-type* cells, leading to tissue overgrowth. Similar mechanisms of non-cell-autonomous tissue growth or tumorigenesis induced by senescent secretory cells are also observed in other settings in* Drosophila* and in human cancers (reviewed in [28, 53]).

### 4.2. Intertumoural Cooperation

**Figure 4:**
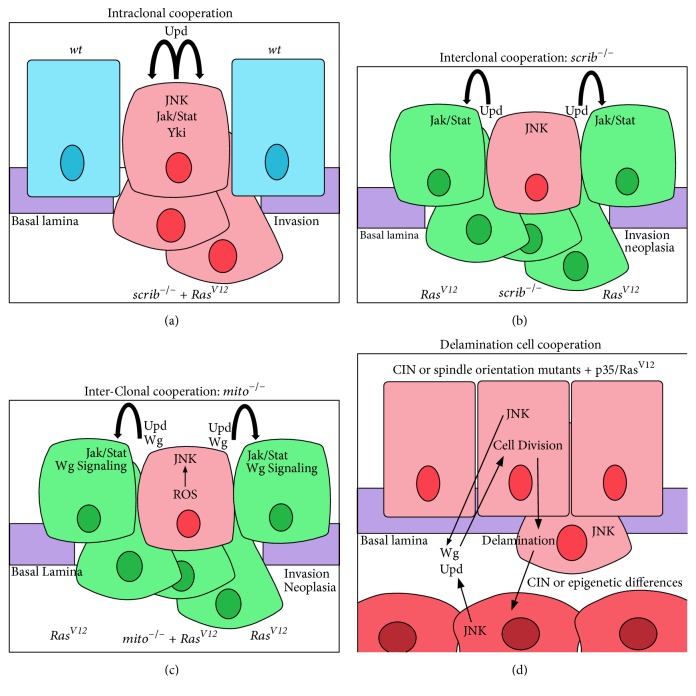
*Different modes of cooperative tumorigenesis*. Examples of different modes of cooperative tumorigenesis. Mutant cells are in pink,* Ras*^*V12*^-expressing cells are in green,* wild-type* cells are in blue, delaminated mutant cells are in dark pink, and the basement membrane (basal lamina) is in purple. (a) Intraclonal cooperation with cell polarity mutants and* Ras*^*V12*^: JNK activation in the tumour cells cooperates with oncogenic Ras signalling to promote tumour overgrowth and invasion. (b) Interclonal cooperation with cell polarity mutants and* Ras*^*V12*^: JNK signalling and Hippo pathway impairment in the* scrib* mutant cells lead to the production of Upd, which induces Dome-Jak-Stat signalling in the surrounding* Ras*^*V12*^-expressing cells, thereby inducing their overgrowth and invasion. (c) Interclonal cooperation with a mitochondrial mutant overexpressing* Ras*^*V12*^ and* Ras*^*V12*^*-*expressing surrounding cells: Upd and Wg are produced by the mitochondrial mutant* Ras*^*V12*^-expressing surrounding cells (see Figure 3(c)), which induce upregulation of Dome-Jak-Stat and Wg signalling, respectively, in the* Ras*^*V12*^ cells to induce their neoplastic overgrowth and invasion. (d) Delaminating cells cooperation: in tumours generated by chromosome instability (CIN) mutants* (rod, bub3,*  and * asp)* or mutants that effect spindle orientation* (scrib, dlg, *and* mud)*, some cells delaminate, resulting in two populations of cells, which in the case of spindle orientation mutants are not genetically different. The delaminated cell population produces the Wg and Upd ligands to upregulate Wg and Dome-Jak-Stat pathways, respectively, in the nondelaminated cells, thereby inducing their proliferation.

Recent studies in* Drosophila* have revealed mechanisms by which cells of different populations can cooperate to generate neoplastic tumours [61, 137–140, 278, 279] (Figure 4, Table 2). Remarkably,* Ras*^*V12*^ cells generated next to* scrib* mutant cells (interclonal), rather than in the same cells (intraclonal, Figure 4(a)), became neoplastically transformed [137] (Figure 4(b)). This occurred by upregulation of JNK signalling and Upd expression in the* scrib*^−^ cells, which induces Dome-Jak-Stat signalling in the neighbouring* Ras*^*V12*^ cells, thereby promoting neoplastic overgrowth. A similar mechanism involving Dome-Jak-Stat signalling, together with Wg signalling, induces neoplastic overgrowth of* Ras*^*V12*^ cells when generated next to mitochondrial respiratory chain gene mutant cells that were also overexpressing* Ras*^*V12*^ [138] (Figure 4(c)). The* Ras*^*V12*^-expressing mitochondrial gene mutant cells exhibit properties of cellular senescence and acquire a secretory phenotype, through ROS, p53, and JNK upregulation, leading to JNK signalling amplification, similar to that which occurs in response to cellular stress [280], which leads to the transcriptional upregulation of Upd and Wg expression [139].

Aneuploidy, generated by mutations in spindle-assembly checkpoint (SAC) genes* (asp*,* rod*, and* bub3)* together with blocking apoptosis, also results in cooperative tumorigenesis involving tumour heterogeneity [61, 140, 142, 278] (Figure 4(d)). In this case, CIN induces metabolic stress leading to ROS production, which, via the JNKKK, Ask, activates JNK, leading to cell delamination and basal extrusion of the aneuploid cells. JNK activity also induces Upd and Wg upregulation and secretion from the aneuploid cells, which act on the mutant epithelial cell population to drive their proliferation through the Dome-Jak-Stat and Wg signalling pathways, respectively [61, 140, 142]. The delaminated cells (mesenchymal-like cells) are unable to proliferate but contribute to tumorigenesis by secreting Upd and Wg. Thus, two cellular populations, with the same original genotype, one epithelial and the other mesenchymal-like (which contains aneuploid cells), cooperate to generate the neoplastic tumour. Strikingly, spindle orientation defective mutants, such as* mud* (an ortholog of mammalian* Numa*, which is important for the localization of Dynein/Dynactin motor proteins in spindle orientation), or the cell polarity mutants,* scrib* and* dlg*, also lead to the generation of two cellular populations, which, upon blocking cell death, cooperate to promote neoplastic tumour formation [61, 141]. In this case, spindle misorientation causes the extrusion of cells from the epithelium, where they lose cell-cell adhesion and their epithelial morphology. Thus, these two populations are not genetically different, although, due to the loss of cell polarity and altered signalling pathways in the delaminated cells, they are likely to have different transcriptomes. Mechanistically, cooperation involves induction of JNK signalling, through a Rho1-Wallenda pathway leading to transcriptional upregulation of Upd and Wg [61]. Additionally, knockdown of dosage compensation genes (*msl1* or* msl2* in males, or* Sxl* in females), which result in genome-wide expression changes on the X chromosome similar to aneuploidy, also result in tumour heterogeneity-induced cooperative tumorigenesis when cell death is blocked [142] (Table 2). Here ROS and JNK signalling are induced and the delaminating cells upregulate MMP1. Thus, dosage compensatory gene mutants, when cell death is blocked, show a similar mechanism to CIN, due to SAC gene knockdown, in inducing tumour heterogeneity.

In summary, the analysis of cooperative tumorigenesis in* Drosophila* has revealed several different mechanisms by which tumour heterogeneity is generated and elucidated the mechanism by which two populations of cells can cooperate in promoting neoplastic tumours. Since heterogeneity is a common phenomenon and an important factor in human cancer (reviewed in [53, 261–264]), the findings in* Drosophila* may provide insight into understanding how heterogeneity arises and contributes to human cancer progression.

## 5. Conclusions and Future Perspectives

We have highlighted in this review how damaged cells are recognized and eliminated in epithelial tissue (cell competition) and the dreadful consequences of the failure of these surveillance mechanisms or of cell death. The persistence of damaged cells, by blocking cell death or by the activation of various oncogenes, drives hyperplastic or neoplastic tumorigenesis by various mechanisms. In cooperative tumorigenesis, which can occur in a myriad of ways, signalling pathways are deregulated to promote tumorigenesis, of which the JNK, Upd (IL6)-Dome-Jak-Stat, and Wg pathways are highly prominent. Interestingly, tissue regeneration also requires these pathways [281], suggesting that normal tissue repair mechanisms are usurped during neoplastic tumorigenesis. As with human cancer, the interaction of the epithelial tumours with their microenvironment plays an important role in neoplastic tumour development in* Drosophila* models. Moreover, non-cell-autonomous cell proliferation induced by the mutant cells affects the surrounding* wild-type* tissues, causing aberrant tissue overgrowth when mutant cell apoptosis is blocked. Additionally,* Drosophila* studies have revealed how tumour heterogeneity arises and has delineated novel mechanisms by which different cell populations are involved in cooperative tumorigenesis. These include the interplay between* scrib* mutant cells juxtaposed to oncogenic Ras-expressing cells and the senescence-induced secretory phenotypes generated by mitochondrial respiratory chain mutations together with oncogenic Ras, where each induces neoplastic tumours non-cell-autonomously. Furthermore, cell polarity, spindle orientation, and spindle-assembly checkpoint mutants, which cause delamination of cells, together with blockage of cell death or the activation of oncogenic pathways, lead to tumour heterogeneity and the crosstalk between two cellular populations to promote neoplastic tumorigenesis. Taken together,* Drosophila* studies have revealed novel cooperative gene interactions in tumorigenesis and the mechanisms by which this occurs, which is of relevance to human cancer. In the Omics age of human cancer research, the plethora of information that is being generated is often difficult to fathom, and functional studies are required to reveal the important cancer drivers and how they cooperate. Due to its sophisticated genetics,* Drosophila* will continue to play an important role in revealing the function of cancer-causing genes* in vivo* and elucidating their mechanisms of action in cooperative tumorigenesis. Moreover,* Drosophila* is now emerging as a highly suitable model organism for the discovery of anticancer compounds against various cancer types, which can be then developed for clinical use, with reduced need for animal models (reviewed in [3, 10, 13, 14, 282, 283]). Thus, in the new age of pharmacogenetics,* Drosophila* will continue to play a fruitful role in elucidating new cooperative gene interactions in cancer and identifying anticancer compounds that then can be harnessed for anticancer therapy.

## Figures and Tables

**Table 1:** Cooperating genes in *Drosophila* tumorigenesis.

Cell-autonomous cooperative tumorigenesis		
1st mutation/mechanism	2nd mutation/mechanism	Phenotype/references
*Cell polarity gene perturbations*		

*Loss of function in apicobasal polarity regulators* Results in cell polarity loss, JNK activation, mild Hippo pathway impairment		Neoplastic overgrowth in whole tissue context and cell polarity loss and apoptosis in clonal context (reviewed in [19])

*Scribble (scrib, dlg, lgl) and Par and Crb polarity module gene loss of function* Scribble module loss of function phenotypes dependent on aPKC activation	*Ras* ^*V12*^ overexpression Dependent on ROS production, TNF (Egr)-JNK signalling, caspase (Dronc) activity Dependent on impairment of Hippo signalling Dependent on PI3K signalling and glutamate utilization	Invasive neoplastic tumours of the larval eye-antennal epithelium [23, 48, 57, 60, 65–68]

*scrib *loss of function,* aPKC-CA *overexpression, *crb *overexpression	Inhibition of JNK signalling	Neoplastic tumour overgrowth in eye-antennal epithelium [66]

*lgl *or* scrib* loss of function Results in JNK activation	*Ras* ^*V12*^ overexpression Requires Hippo pathway impairment	Neoplastic tumours in the larval wing epithelium [49]

*scrib* loss of function Results in JNK activation	*Notch* ^*intra (Act)*^ overexpression	Invasive neoplastic tumours in the larval eye-antennal epithelium [23, 66, 69]

*scrib* loss of function Results in JNK activation	Abrupt (BTB-POZ Zn finger transcription factor) overexpression Results in JNK activation Results in Hippo pathway impairment Results in downregulation of differentiation and Ecdysone response genes	Invasive neoplastic tumours in the eye-antennal and wing epithelial tissues [70]

*scrib* loss of function	Taiman (Ecdysone coactivator) Results in reduced differentiation	Invasive neoplastic tumours in the eye-antennal and wing epithelial tissues [70]

*scrib* loss of function Results in JNK activation	Slit-Robo2-Ena loss of function	Overgrown tumours in the eye-antennal epithelial tissues [45]

*scrib* loss of function Results in JNK activation	Slit-Robo overexpression Requires Ena Results in JNK activation and activation of a positive feedback loop	Excessive extrusion and luminal tumour overgrowth in larval eye-antennal epithelial tissues [45]

*lgl* loss of function Results in Hippo pathway impairment	Inhibition of JNK signalling	Invasive neoplastic tumours of the larval/pupal eye neural-epithelium [71]

*lgl* loss of function	Myc overexpression	Invasive neoplastic tumours of the larval wing epithelium [50]

*lgl* loss of function	Hippo pathway impairment	Neoplastic tumours of the larval wing epithelium [49]

*lgl* loss of function	*Notch* ^*intra (Act)*^ overexpression	Neoplastic tumours of the larval wing epithelium [72]

*scrib, dlg *or *lgl *depletion	*cno *mutants Results in activation of Ras-MAPK signalling	Enhanced neoplastic tumours of the antennal epithelium [73]

*Par-1 overexpression* Results in cell polarity loss and Hippo pathway impairment		Eye-antennal and wing tissue overgrowth [74]

Par-1 overexpression	*Notch* ^*intra (Act)*^ overexpression	Hyperplastic eye-antennal epithelium [75]

*Actin cytoskeletal regulators *		

Activation of Actin cytoskeletal regulators *Rac1, RhoGEF2, Pbl, Rho*^*V14*^*, Rho1, Rok*^*CAT*^*, sqh*^*EE*^ Results in activation of JNK signalling and cell morphology changes	Ras^V12^ (Raf gain-of-function) overexpression	Invasive neoplastic tumours of the larval eye-antennal epithelium [76, 77]

RhoGEF2 overexpression Results in JNK activation and cell morphology changes	Abrupt (BTB-POZ Zn finger transcription factor) overexpression Results in reduced expression of differentiation gene, Dac	Neoplastic tumours of the larval eye-antennal epithelial tissue [78]

Src64B overexpression Results in JNK activation and cell morphology changes	Blocking JNK Rac1-Dia, Ras-MAPK, Hippo pathway impairment	Eye-antennal epithelial tissue overgrowth [79]

*csk* loss of function (Src activation) Depends on Actin cytoskeleton regulators, JNK activation, STAT activation, Hippo pathway impairment, Wingless (Wnt) expression/signalling and insulin-PI3K signalling	Ras^V12^ overexpression Promotes cell proliferation and survival	Invasive neoplastic tumours of the larval eye-antennal epithelium [80–85]

Src42A or Src64B overexpression Results in Egr independent activation of JNK and Jak-Stat signalling	Notch^intra (Act)^ overexpression	Neoplastic tumours of the larval eye-antennal and wing epithelium [86]

Src64B overexpression Results in cell morphology changes	Abrupt overexpression Reduces expression of differentiation gene and Dac and Dll	Neoplastic tumours of the larval eye-antennal epithelial tissue [78]

Troponin I overexpression	*Ras* ^*V12*^ overexpression *Notch*^*intra (Act)*^ overexpression *lgl* mutant *Ras*^*V12*^ overexpression	Tumour overgrowth or neoplastic tumour overgrowth in wing epithelial tissue [87]

*Signalling pathway deregulation*		

*Ras* ^*V12*^ * overexpression* Results in tissue overgrowth, which depends upon EGF-EGFR activation and Arf6 mediated Hedgehog signalling		Eye-antennal and wing epithelial tissue overgrowth [88]

	TNF-JNK signalling	Invasive neoplastic tumours in the larval eye-antennal epithelium [76, 89–91]

	Immune signalling and activation of JNK	Invasive neoplastic tumours of the adult hindgut epithelium [92]

	Ben/dUev1a E2 ubiquitin ligase overexpression Results in JNK activation (via binding Traf2)	Invasive neoplastic tumours in the larval eye-antennal epithelium [93–95]

	*sds22* (PP1) loss of function Results in cell morphology/polarity loss Results in Myosin II, JNK activation	Invasive neoplastic tumours of the larval eye-antennal epithelium [96]

	PP6 phosphatase (FMT, PpV) knock down Results in Tak1-JNK activation	Invasive neoplastic tumours of the eye-antennal epithelium [97]

	Infection/inflammation Results in Imd-dTab2-dTak1-JNK signalling and MMP1 expression	Hindgut epithelial tumour invasion [92, 98]

	Impaired Hippo pathway signalling Results in upregulation of Ras pathway genes, Upd-Jak-Stat signalling	Eye-antennal and wing tissue overgrowth [65, 99]

	Lysosomal protein loss of function—*deep orange, carnation, vps16A*	Invasive neoplastic tumours of the larval eye-antennal epithelium [100]

	Autophagy loss of function—e.g., *Atg8a, Atg7, Atg9, Atg1, Atg13, Syx17* Requires ROS and JNK upregulation	Invasive neoplastic tumours of the larval eye-antennal epithelium [101]

	Chromosome remodelling complex mutation *polyhomeotic* Depends on ectopic Notch activation	Invasive neoplastic tumours of the larval eye-antennal epithelium [102]

	Chinmo (BTB-POZ Zn finger transcription factor) overexpression	Overgrown tumours in the eye-antennal epithelial tissues [69]

	Fruitless (BTB-POZ Zn finger transcription factor) overexpression	Overgrown tumours in the eye-antennal epithelial tissues [69]

	*PTEN* knockdown (Elevated PI3K signalling)	Larval-Pupal tracheal epithelial tissue invasive tumours [103]

	*apc* (Wingless/Wnt) signalling	Adult midgut epithelial tissue overgrowth [104, 105]

	*p53, apc, pten *knockdown *dSmad4*, *apc, pten *knockdown	Adult hindgut epithelial tissue invasive tumours [106]

	*pico (MRL) *overexpression *chickadee *(Profilin) overexpression *mal *(SRF cofactor gene) overexpression Requires JNK-MMP1 activity	Glial cell overgrowth and invasion [107]

*EGFR activation/overexpression* Depends on Ras and Hh signalling		Eye-antennal and wing epithelial tissue overgrowth [88]

	*fat *loss of function (Hippo pathway impairment)	Eye-antennal and wing epithelial tissue overgrowth [108]

	*bantam* micro-RNA expression Results in downregulation of Socs36E Leads to increased Jak-Stat signalling	Invasive overgrowth of the larval wing epithelium [109]

	*miR-10 *or* miR-375 *Micro-RNA expression Results in downregulation of Psq transcription factor	Invasive overgrowth of the larval eye-antennal and wing epithelium [52]

	*miR-8* Micro-RNA expression Results in downregulation of Peanut protein expression, cytokinesis blockage, formation of polyploid cells	Invasive overgrowth of the larval wing epithelium [110]

	PI3K pathway activation Requires Tor, Sin1, Rictor, Myc, Cyclin D-Cdk4, Rb-E2F and Cdc25 Requires RIOK1, RIOK2	Glia cell invasive brain tumours and eye neural-epithelium tumours [111–113]

*Notch* ^*intra (Act)*^ */Delta overexpression* Results in tissue overgrowth		Eye-antennal and wing tissue overgrowth (reviewed by [114])

Notch^intra (Act)^ overexpression	Mef2 overexpression	Invasive neoplastic tumours of the larval eye neural-epithelium [115]

Notch^intra (Act)^ overexpression	Chinmo (BTB-POZ Zn finger transcription factor) overexpression	Overgrown tumours in the eye-antennal epithelial tissues [69]

Notch^intra (Act)^ overexpression	Fruitless (BTB-POZ Zn finger transcription factor) overexpression	Overgrown tumours in the eye-antennal epithelial tissues [69]

Delta overexpression	Overexpression of transcription factors Psq/Lola (*eyeful* model)	Invasive tumours larval/pupal eye neural-epithelium, which are capable of differentiation to express ELAV [116]

Delta overexpression	Overexpression of Akt or PI3K (Dp110)	Invasive tumours larval/pupal eye neural-epithelium, which are capable of differentiation (ELAV expression) [117]

Delta overexpression Results in repression of *boi *gene expression and reduced Hedgehog signalling	Overexpression of *mir-7 *micro-RNA Results in downregulation of *ihog* translation and reduced Hedgehog signalling	Eye-antennal disc overgrowth and invasive cells cable of differentiation [118]

Delta overexpression	Overexpression of *Zfh1* (Zeb1 family transcription factor gene)	Invasive tumours larval/pupal eye neural-epithelium, which are capable differentiation to express ELAV [119]

Delta overexpression Delta with Pipsqueak/Lola overexpression (*eyeful* model)	Knockdown of* atonal* (transcription factor gene) Results in reduced JNK signalling	Invasive tumours larval/pupal eye neural-epithelium, which are capable differentiation to express ELAV [120]

Delta overexpression Delta with Pipsqueak/Lola overexpression (*eyeful *model)	Knockdown of *cut* (transcription factor gene) Results in increased *reaper* expression and elevated PI3K-Akt signalling	Invasive tumours in larval/pupal eye-antennal epithelium, which are capable differentiation to express ELAV [121]

*Hippo pathway impairment (Yki overexpression)* Results in increased tissue growth through upregulation of cell growth (Myc), proliferation (CycE) and antiapoptotic genes (Diap1), elevation of Upd-Jak-Stat signalling		Increased tissue growth (reviewed by [122])

	BAP (Brahma) complex knockdown (*brm, Snr1, mor, Bap111, osa*) Results in upregulation of Wingless (Wnt) and Dpp signalling	Neoplastic tumour overgrowth in larval wing epithelial tissue [123]

	Taiman (Ecdysone Receptor coactivator) overexpression Results in expression of germline stem cell factors	Hyperplastic tumour overgrowth in larval wing epithelial tissue [124]

*Guidance receptors*		

*Frazzled (Dcc) loss of function and expression of the Caspase inhibitor P35* Results in an invasive phenotype in eye epithelial cells, but cells can differentiate	Inhibition of JNK signalling Requires Rho1	Invasive, but differentiated, tumours in larval/pupal eye-antennal epithelial tissues [125]

*Mitotic checkpoint, chromosome instability, DNA damage repair genes*		

*Nek2 (centrosomal kinase) overexpression*	*Ret* ^*MEN2B*^ overexpression—elevated Ras, PI3K, Src, JNK signalling *Csk*^−^* Ras*^*V12*^ Results in Rac1, Rho1, Wg signalling and elevated expression of Diap1, MMP1 Results in PI3K signalling	Invasive tumours in larval eye-antennal epithelial tissue [126]

*bub3 knockdown* Results in aneuploidy	*p35* overexpression to block effector caspase activity	Neoplastic overgrowth of wing epithelial cells [127]

*DNA repair or DNA damage checkpoint mutants* Depletion of *okra* (*DmRAD54*) or *spnA* (*DmRAD51*) (Homologous recombination of DNA double strand-breaks in G2) *grp* (*chk1*) and *mei-41* (*ATR*) knockdown (DNA damage checkpoint)	Ionizing irradiation and *p35* overexpression Results in JNK activation, which leads to MMP1 and Wg upregulation	Overgrowth and cell delamination/migration in the wing epithelial tissue [128]

**Table 2:** Tumour-wild-type tissue and intertumoural interactions.

Non-cell autonomous overgrowth/tumorigenesis		
1st mutation/mechanism	2nd mutation/mechanism	Phenotype/references
*hid/rpr overexpression*	p35 expression (blocks effector caspase activity) generates “undead cells”	Eye-antennal or wing epithelial tissue non-cell autonomous overgrowth [59, 129–134]
Initiator caspase (Dronc) activation	Dependent on ROS production, JNK activation, Dpp, Wg upregulation/secretion	

*ptc mutant*	*ark* (*apaf1*) mutant	Eye-antennal and wing epithelial tissue non-cell autonomous overgrowth [135]
Hh pathway upregulation	Results in upregulation of Dpp secretion from mutant cells and elevated Dpp signalling and Yki activity in the *wild-type* cells	

*Raf* ^*GOF*^ * overexpression*	Strong activation of JNK (*hep*^*ACT*^)	Non-cell autonomous overgrowth and morphology changes of eye-antennal epithelial tissue [136]

*Rac1 overexpression*	Abrupt (BTB-POZ Zn finger transcription factor)	Eye-antennal epithelial tissue non-cell autonomous overgrowth [78]

*Intertumoural cooperation*		

*Ras* ^*V12*^ * overexpression*	*scrib* mutant cells next to *Ras*^*V12*^ overexpressing cells	Invasive neoplastic tumours of the larval eye neural-epithelium [137]
	Activation of JNK and Upd upregulation and Jak-Stat signalling	

	Mitochondrial dysfunction in *Ras*^*V12*^ overexpressing cells next to *Ras*^*V12*^ overexpressing cells	Invasive neoplastic tumours of the larval eye neural-epithelium [138, 139]
	Results in ROS production, upregulation of JNK, deregulation of Hippo, secretion of Wg and Upd	

*Chromosome instability*	Cell death blockage (*p35* expression)	Invasive tumours in the wing epithelium [61, 140]
Induced by spindle assembly or spindle-assembly checkpoint mutants leading to cell delamination (*rod*, *bub3*, *asp*)	Results in Metabolic stress, ROS induced-JNK activation in epithelial cells promoting cell delamination	
	Results in Secretion of Wg, Upd from delaminated cells, promoting the proliferation of epithelial tumour cells	

*Spindle orientation defects*	Cell death blockage (*p35 *expression) with *mud* knockdown	Invasive tumours in the eye-antennal or wing epithelium [61, 141, 142]
Due to mutants/knockdown of genes involved in spindle alignment (*mud*, *scrib, dlg*) leading to cell delamination	*Ras* ^*V12*^ or *p35* expression with *scrib* or *dlg*	
	Results in Rho1-Wnd induced JNK activation in epithelial cells promoting cell delamination	
	Results in secretion of Wg, Upd from delaminated cells, promoting the proliferation of epithelial tumour cells	

*Dosage compensation mechanism mutants*	p35 overexpression or deletion of *rpr, hid, grim* to block cell death	Invasive tumours in the wing epithelium [142]
Knockdown of *msl1* or *msl2* in males or *Sxl* in females	Results in ROS induced-JNK activation	
	Results in MMP1 expression in delaminating cells	
